# Molecular pathogenesis of desmoid tumor and the role of γ-secretase inhibition

**DOI:** 10.1038/s41698-022-00308-1

**Published:** 2022-09-06

**Authors:** Noah Federman

**Affiliations:** Departments of Pediatrics and Orthopedics, UCLA Jonsson Comprehensive Cancer Center, UCLA David Geffen School of Medicine, 10833 Le Conte Ave., Los Angeles, CA 90095 USA

**Keywords:** Oncogenesis, Paediatric cancer, Molecular medicine, Sarcoma

## Abstract

Desmoid tumor (DT) is a rare, soft tissue neoplasm associated with an unpredictable clinical course. Although lacking metastatic potential, DT is often locally aggressive and invasive, causing significant morbidity. Both sporadic DT and familial adenomatous polyposis (FAP)-associated DT are linked to constitutive activation of the Wnt signaling pathway with mutations in the β-catenin oncogene *CTNNB1* or the tumor suppressor gene *APC*, respectively. Cross-talk between the Notch and Wnt pathways, as well as activation of the Notch pathway resulting from dysregulation of the Wnt pathway, suggest a possible therapeutic target for DT. Due to the role γ-secretase plays in Notch signaling through cleavage of the Notch intracellular domain (with subsequent translocation to the nucleus to activate gene transcription), γ-secretase inhibitors (GSIs) have emerged as a potential treatment for DT. Two GSIs, nirogacestat (PF-03084014) and AL102 are in later-stage clinical development; nirogacestat is being evaluated in a phase 3, randomized, placebo-controlled trial while AL102 is being evaluated in a phase 2/3, dose-finding (part A) and placebo-controlled (part B) trial. This review summarizes current understanding of the molecular pathogenesis of DT focusing on dysregulation of the Wnt signaling pathway, crosstalk with the Notch pathway, and the potential therapeutic role for GSIs in DT.

## Introduction

Desmoid tumor (DT), also known as aggressive fibromatosis or desmoid-type fibromatosis, is a monoclonal proliferation of myofibroblasts^1^ that arises in the deep soft tissues and may occur in the abdominal and chest walls, mesenteric root, or extremities^2,3^. Evidence from animal models and cell transplantation data suggests that mesenchymal stem cells are the neoplastic cell of origin in DT^4,5^. DT occurs rarely (accounting for fewer than 3% of soft tissue tumors)^6^, with a reported incidence of 3–5 cases per million population per year^1,7–12^.

Onset of DT has been reported from infancy through adulthood, although it occurs most commonly between the ages of 15 and 60 years (with peak incidence between 30–40 years of age) and 2–3 times more frequently in women than men^2,10,13^. It has been observed that DT may occur in association with pregnancy and with the use of estrogen-containing oral contraceptives^14^; and, in some (but not all) patients, to stabilize or regress post-partum and with menopause, theoretically as a result of hormonal changes that occur. Other risk factors include surgery and trauma^14^. Approximately 85–90% of DT cases are sporadic, associated with mutations in the *CTNNB1* gene that encodes β-catenin, while 5–10% of cases arise in the context of familial adenomatous polyposis (FAP) in which there is a germline mutation in the adenomatous polyposis coli gene (*APC*)^1^.

While lacking metastatic potential, the natural history of DT is variable and ranges from an asymptomatic, indolent course to aggressive infiltration of neurovascular structures and vital organs resulting in pain, disfigurement, organ dysfunction, and— rarely—death (usually related to complications such as intestinal obstruction associated with progression of intra-abdominal tumors)^15^. Historically, the primary treatment for DT was surgery, however, high rates of local recurrence and poor functional outcomes following surgery have led to a shift toward nonsurgical approaches, either active surveillance or medical management^2^.

Treatment options and optimal sequencing of these options are rapidly evolving^16^. There is no “one-size fits all” approach to DT patients, and treatment decisions should be made with an experienced multidisciplinary musculoskeletal oncology group together with the patient. Spontaneous regression is well reported in DT and may occur in up to 10–20% of patients^17,18^. Several studies have demonstrated that a period of surveillance (also called “watchful waiting”) can result in long-term avoidance of local and systemic interventions^19^. Thus, nearly all patients with asymptomatic DT should receive a period of active surveillance. Those patients who have symptomatic DT, progression on surveillance, or anatomical sites (e.g., head/neck, mesentery) that could be devastating with progression will warrant therapy.

Locoregional treatments that have shown some benefit in managing DT include radiation therapy, cryoablation, and high-intensity focused ultrasound^20^. Although no medications have as yet received regulatory approval for the treatment of DT^21^ a number of alternatives have been investigated, including hormonal therapy, non-steroidal anti-inflammatory drugs (NSAIDs), chemotherapy, and small molecule therapies such as tyrosine kinase inhibitors (TKIs)^1^. Estrogen has long been thought to modulate DT^22^, but evidence for the effectiveness of anti-estrogen therapies is limited to case series and single-arm trials. Consequently, treatment guidelines no longer recommend hormonal therapies for DT^1,21^. The rationale for the use of NSAIDs in DT was based on the observation that cyclooxygenase-2 (COX-2) is overexpressed in these tumors^23^, and several NSAIDs (some in combination with hormonal therapy) have been studied in open-label and observational trials^24–26^. To date, however, there have been no randomized, prospective studies of NSAIDs as disease-modifying agents in DT, and current treatment guidelines recommend their use only for pain relief^21^.

As with hormonal therapy and NSAIDs, evidence for the effectiveness of cytotoxic chemotherapy in DT comes from retrospective and prospective, non-randomized studies^1^. A recent review of clinical outcomes in DT patients treated with chemotherapy (either low-dose methotrexate plus vinblastine or vinorelbine or a conventional anthracycline-containing regimen) showed disease control rates of 64–100%^27^, however, these agents may be associated with hematologic toxicities. While the precise mechanism of action of TKIs in DT is not known, their activity to inhibit vascular endothelial growth factor receptors (VEGFRs) and/or platelet-derived growth factor receptors (PDGFRs)^28^ may interfere with DT growth and progression. Based on evidence from prospective trials (including a phase 3 randomized, double-blind, placebo-controlled trial of sorafenib)^17,29–34^ TKIs are currently recommended as a systemic treatment option for patients with progressive DT, although their safety and tolerability for long-term use have yet to be fully assessed^1,27^.

The choice and sequence of therapy, including treatment with systemic agents, depends on individual patient characteristics such as age and comorbidities, as well as risk factors for recurrence such as location. For example, smaller tumors that are easily resectable in the abdominal wall or extremity will usually undergo surgery followed by observation, while intrabdominal DT is rarely amenable to surgery and/or radiation therapy without major potential morbidity, and therefore will be treated systemically. With respect to systemic therapy, the Desmoid Tumor Working Group recommends following a model that considers factors including response rate, ease of administration, and expected toxicity associated with a particular agent^1^, progressing from less to more toxic treatments unless disease severity warrants more aggressive intervention.

More recently, γ-secretase inhibitors (GSIs) have been investigated for the treatment of DT, based on their mechanism of action to inhibit the Notch signaling pathway^35^. Notch is thought to engage in cross-talk with the Wnt/β-catenin signaling pathway^36,37^, which is constitutively activated in DT^38^. In addition, Notch target genes have been shown to be overexpressed in DT^35^.

This review will describe the Wnt and Notch signaling pathways, the evidence for their dysregulation and crosstalk in DT, and the current state of development of therapeutic agents for DT targeting these pathways, with a particular focus on GSIs.

## The Wnt signaling pathway

**Fig. 1:**
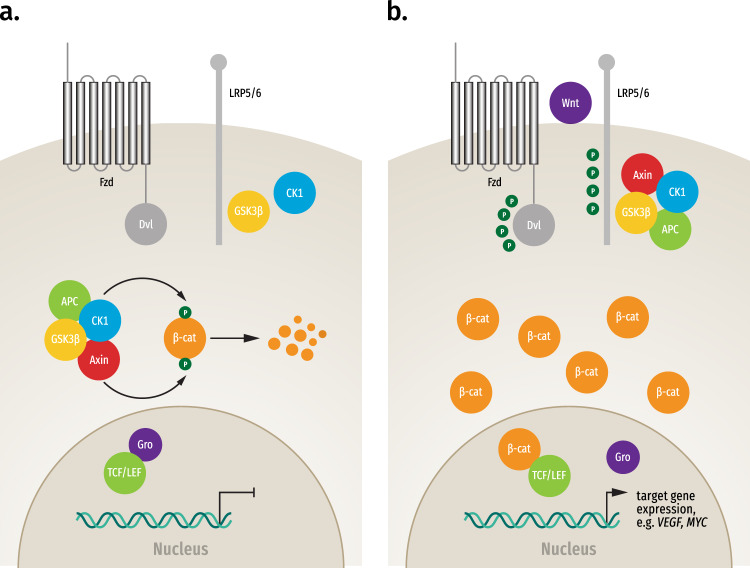
Wnt/β-catenin signaling pathway. **a** In the absence of Wnt ligands, β-catenin (β-cat) in the cytosol is bound and processed by a destruction complex, comprising Axin, adenomatous polyposis coli (APC), glycogen synthase kinase 3β (GSK3β), and Casein kinase 1 (CK1). β-cat is sequentially phosphorylated (P) by CK1 and GSK3β and targeted for degradation, maintaining low levels of cytosolic β-cat. In the absence of Wnt signaling, the T-cell factor/lymphoid enhancer factor (TCF/LEF) family of transcription factors interact with Groucho (Gro) proteins and together act to repress gene expression. **b** In the presence of Wnt ligands, extracellular Wnt binds to Frizzled (Fzd) and low-density lipoprotein receptor-related proteins 5 and 6 (LRP5/6) at the cell membrane, inducing phosphorylation of LRP5/6. Subsequently, Disheveled (Dvl) and Axin are recruited to the membrane, thereby inactivating the destruction complex. As a result, β-cat accumulates in the cytosol and translocates to the nucleus where it binds with TCF/LEF, displaces Gro, and activates expression of target genes.

The Wnt family of proteins is implicated in many cellular functions, including organ formation, stem cell renewal, and cell survival^39^. The highly conserved Wnt signaling pathway regulates both cytosolic and nuclear levels of β-catenin^40^, and canonical Wnt/β-catenin signaling is one of the key cascades regulating embryogenesis and tissue homeostasis^41^. In the absence of Wnt ligands, β-catenin molecules present in the cytosol are bound and processed by a destruction complex formed by the scaffolding proteins Axin and APC, and the kinases glycogen synthase kinase 3β (GSK3β) and Casein kinase 1 (CK1)^42^. Once β-catenin is bound to the destruction complex, it is sequentially phosphorylated by CK1α and GSK3β^43,44^. Phosphorylated β-catenin interacts with the E3 ubiquitin ligase β-transducin repeat-containing protein (β-TrCP) that targets it for proteasomal degradation, thereby maintaining cytoplasmic concentration of β-catenin at low levels and preventing its translocation to the nucleus^45,46^. In the absence of Wnt/β-catenin signaling, the T-cell factor/lymphoid enhancer factor (TCF/LEF) family of transcription factors in the nucleus interact with Groucho proteins and together act as transcriptional repressors, inhibiting the transcription of several genes involved with cellular growth^47^ (Fig. 1a).

In the presence of Wnt ligands, however, a receptor complex containing Frizzled (Fzd) and low-density lipoprotein receptor-related proteins 5 and 6 (LRP5/6) is formed at the plasma membrane, inducing the phosphorylation of LRP5/6 by GSK3β and priming a second phosphorylation by CK1α^48,49^. Subsequently, both Disheveled (Dvl) and Axin are recruited to the membrane, with Dvl interacting with the C-terminal tail of the Fzd protein and Axin with the hyperphosphorylated LRP5/6^50,51^. As a result, Axin is sequestered away from the destruction complex, impairing the phosphorylation of β-catenin, allowing cytoplasmic β-catenin to accumulate and translocate to the nucleus where it binds with the TCF/LEF family of transcription factors, displaces Groucho, and recruits transcriptional co-activators, leading to expression of specific target genes associated with induction of several processes, such as cell proliferation and survival^52,53^ (Fig. 1b).

Dysregulation of the Wnt signaling pathway has been reported in a number of cancers, including breast cancer, colorectal carcinoma, hepatocellular carcinoma, pancreatic ductal adenocarcinoma, and non-small cell lung cancer^54^.

As previously noted, the Wnt pathway plays a key role in DT pathogenesis, with constitutive activation caused by mutations in the β-catenin oncogene *CTNNB1* in most sporadic cases of DT^55^, or a germline mutation in *APC* (which regulates β-catenin degradation) in cases associated with FAP^56^. Approximately 85–90% of sporadic cases of DT show activating mutations in the N-terminal region of *CTNNB1* (all of them in exon 3)^57^, making β-catenin more resistant to proteolytic degradation, leading to cytoplasmic accumulation of the protein and its subsequent translocation to the nucleus. Mutations at T41A and S45F of the *CTNNB1* gene are the most common in DT, accounting for roughly 55% and 35% of cases, respectively. S45P is the third most common mutation at ~10%. Very rare missense mutations and deletions affecting codons 32–49 have been observed as well^58,59^. In ~5–10% of patients, DT results from germline (i.e., in FAP) or sporadic loss-of-function mutations in the *APC* tumor suppressor gene. Loss of *APC* leads to activation and accumulation of β-catenin because APC functions as a negative regulator of β-catenin stability.

Dysregulation of the Wnt pathway in DT leads to overexpression of Wnt genes involved in proliferation and fibrosis, such as *ADAM12*, *Fap-1α*, *WISP1*, and *SOX11*^60^, as well as genes such as *VEGF*, which is involved in the regulation of angiogenesis, and *COX2*, which initiates activation of growth factor receptors, such as the PDGFRs α and ß (PDGF-α and PDGF-ß)^61,62^. Despite clear evidence for the role of Wnt signaling dysregulation in DT, there are substantial challenges to identifying therapeutic targets in the Wnt pathway, particularly in finding agents that are efficacious without being deleterious to the system of normal somatic stem cell function in cellular repair and tissue homeostasis^62^. The Wnt pathway, however, may cross-talk with the Notch pathway^36,37,63–65^, providing alternative potential therapeutic targets in DT.

## The Notch signaling pathway

Like the Wnt pathway, the Notch signaling pathway is highly conserved evolutionarily, and plays an important role in cell development and differentiation, serving key functions ranging from embryonic development to adult homeostasis^66^. The Notch pathway includes 4 receptors (Notch1-4) and at least 5 ligands (Jagged1 [JAG1], JAG2, delta-like 1 [DLL1], DLL3 and DLL4)^67^. The Notch transmembrane receptor glycoproteins function as membrane-bound transcription factors that regulate critical cellular functions including differentiation, cell fate determination, proliferation, self-renewal, and survival^68^.

**Fig. 2:**
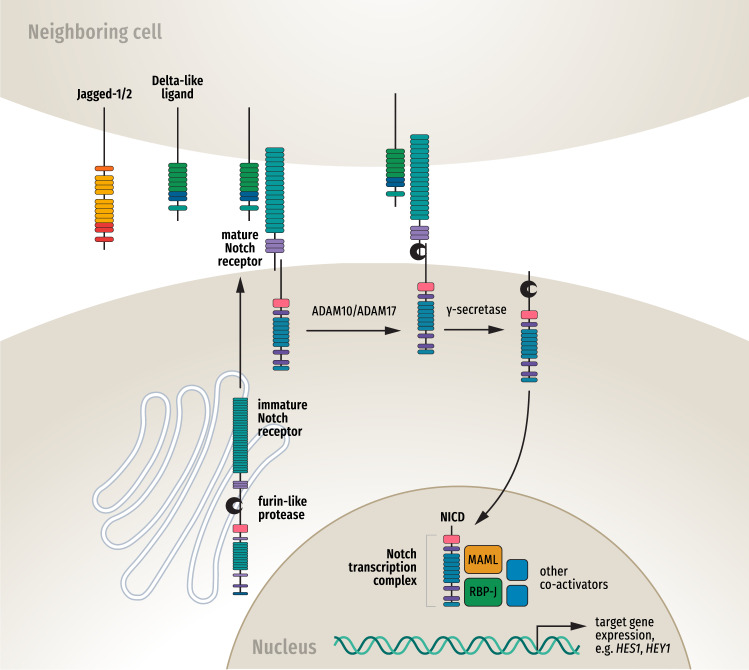
Notch signaling pathway. The immature Notch receptor is processed in the Golgi network where a furin-like protease cleaves it to create the mature Notch receptor, comprising the large extracellular domain linked to the smaller transmembrane domain and intracellular domain (NICD). Notch-specific ligands Jagged-1/2 or Delta-like ligand-1/3/4 bind the extracellular domain and activate the sequential cleavage of the Notch protein by a member of the disintegrin and metalloprotease family (ADAM 10/17) and γ-secretase, releasing the NICD which then translocates to the nucleus. In the nucleus NICD forms a transcription complex with the recombination signal binding protein for immunoglobulin kappa J region (RBP-J) transcription factors, the mastermind-like (MAML) proteins and other coactivators to stimulate expression of Notch target genes including the Hairy Enhancer of Split (*HES*) and HES-related proteins (*HEY*).

The Notch receptor contains three domains: the extracellular domain (NECD), the transmembrane domain (NTMD), and the intracellular domain (NICD)^68^. Following ligand binding, the Notch receptor protein undergoes sequential cleavage by a member of the disintegrin and metalloprotease family (ADAM10/17)^69^ and γ-secretase enzymes^70^ (Fig. 2).

The NECD remains bound to the ligand, while the second of these cleavages by γ-secretase occurs within the NTMD and releases the NICD which then translocates to the nucleus^70^. In the nucleus the NICD forms a Notch transcription complex, comprising NICD, the recombination signal binding protein for immunoglobulin kappa J region (RBP-J, also called CBF1/Suppressor of Hairless, and Longevity-Assurance Gene-1 [CSL]) family of transcription factors, and the mastermind-like (MAML) family of proteins to activate transcription of target genes^71^. Notch target genes include transcription factors Hairy Enhancer of Split (*HES*) and HES-related proteins (*HEY*), as well as genes regulating the cell cycle and apoptosis^40^.

Dysregulated Notch signaling is implicated in hematologic malignancies, including lymphoid leukemia, as well as solid tumors of the breast, ovary, lung, pancreas, colon, head and neck, cervix, and kidney^72^. Cross-talk between the Notch and Wnt pathways was first described in *Drosophila*, in which Notch is co-expressed with Wingless (the *Drosophila* homolog of Wnt) and regulates Wingless signaling^73^. Cross-talk between these two pathways has also been observed in cultured cancer cells and in various animal models of disease^63–65^. In addition, it has been shown that β-catenin-mediated upregulation of JAG1 activates Notch signaling in tumors from patients with FAP^37^.

Studies in APC^min^/^+^ mouse colorectal cancer models showed that *HES1*, a Notch target gene, was significantly upregulated^65^. Moreover, it has been shown that the induction of HES1 can also be mediated directly by activated β-catenin signaling in a mouse model with acute APC loss in the adult intestinal epithelium, as well as in a human colorectal cell line with a truncated form of APC that cannot target β-catenin for degradation^64^. These results suggest that dysregulation of the Wnt pathway results in activation of Notch signaling. In DT, evidence for dysregulation of Notch signaling was observed in in vitro studies showing that DT tissues expressed higher levels of Notch1 and its downstream target HES1^4^. Subsequent studies analyzed the expression of Notch pathway components in DT tissues and cell strains, and confirmed increased expression of HES1 in DT versus dermal scar tissue^35^. In addition, exposure of DT cell strains to the GSI nirogacestat (formerly known as PF-03084014) resulted in significant decreases in NICD and HES1 expression, decreased cell migration and invasion, and inhibition of cell growth^35^.

## Molecular targets for pharmacologic treatment of DT

While dysregulation of the Wnt signaling pathway is clearly implicated in the etiology of DT and solid tumor cancers, therapeutic targeting of the Wnt pathway remains challenging due to its biologic importance in maintaining homeostasis of adult tissues^74^. As described previously, the rationale for treatment of DT with NSAIDs and TKIs is based on inhibition of the activity of Wnt target gene products such as COX, VEGFR, and PDGFR.

Tegavivint (formerly BC-2059) is a small molecule inhibitor of the Wnt pathway with potential activity in DT. Tegavivint binds to transducin β-like protein 1 (TBL1), thereby disrupting binding of β-catenin to TBL1 and promoting β-catenin degradation^75^. A phase 1 open-label trial of tegavivint in 24 adult subjects with unresectable, symptomatic or progressive DT was initiated in July 2018 (NCT03459469^76^) and a phase 1/2 trial of tegavivint in children, adolescents, and young adults (ages 1–30 years) with recurrent or refractory solid tumors, including DT, was initiated in October 2021 (NCT04851119^77^).

Ipafricept (formerly OMP-54F28) is a truncated Fzd receptor monoclonal antibody that binds Wnt ligands, thereby inhibiting Wnt signaling. Results from a phase 1 dose escalation study (NCT01608867^78^) in 26 adult patients with advanced solid tumors showed ipafricept was generally well tolerated (most adverse events were grade 1 or 2), and 2 patients with DT had stable disease for >6 months^79^, but to date no further trials of ipafricept in DT have been initiated.

Investigational agents targeting the Notch signaling pathway include biologics (e.g., monoclonal antibodies, chimeric antigen receptor‑modified T cells) that bind to the extracellular region of Notch receptors or ligands, small molecule inhibitors of the Notch transcription complex which block the NICD-dependent transcription of Notch target genes, and GSIs which block release of the NICD, thereby preventing its translocation to the nucleus^80^. While a number of such therapeutics are in various stages of development for the treatment of both solid tumor and hematologic malignancies^80^, to date only the GSIs have been evaluated in DT.

## Inhibition of γ-secretase in DT

γ-secretase is a membrane-bound protease complex consisting of a catalytic subunit named presenilin (PSEN1 and PSEN2) and three other subunits including nicastrin, anterior pharynx defective-1 (APH1A and APH1B) and presenilin enhancer 2 (PEN-2)^81^. As noted, the potential role of cross-talk between the Notch and Wnt signaling pathways, combined with the overexpression of Notch pathway components, provides a mechanistic rationale for targeting Notch inhibition in DT^35^. Inhibiting the proteolytic activity of γ-secretase prevents the release of the NICD and its translocation to the nucleus—the key step for activation of all downstream effects^70^— leading to decreased expression of several Notch target genes, including those in the *HES* family. Several small-molecule inhibitors of γ-secretase have reported activity in DT in case reports, as well as in several phase 1 and 2 trials^82–87^; two GSIs are in later stage clinical development (Table 1).

**Table 1:** Clinical trials of γ-secretase inhibitors in the treatment of desmoid tumor.

GSI	ClinicalTrials.gov Registration	Phase	Status	Diagnosis	Age (yr)	Dose(s) evaluated	n with DT	Objective response
Nirogacestat (PF-03084014)	NCT00878189^86,90^	1	Completed	Advanced solid tumors	≥18	20–330 mg PO BID	9	5 PR, 2 SD
	NCT01981551^85,92^	2	Active/not recruiting	Progressive DT	≥18	150 mg PO BID	17	5 PR, 11 SD
	NCT04195399^93^	2	Recruiting	Progressive DT	1–18	90 mg/m^2^ PO BID	≈35	TBD
	NCT03785964^94^	3	Active/not recruiting	Progressive DT	≥18	150 mg PO BID	≈142	TBD
AL102 (BMS-986115)	NCT01986218^82,99^	1	Terminated	Advanced solid tumors	≥18	0.3–2 mg PO QD 2–8 mg PO BIW	1	1 SD
	NCT04871282^100^	2/3	Recruiting	Progressive DT	≥12	1.2 mg PO QD 2–4 mg PO BIW	≈192	TBD
AL101 (BMS-906024)	NCT01292655^97,98^	1	Completed	Advanced solid tumors	≥18	0.3–8.4 mg IV QW 4–6 mg IV Q2W	3	2 PR, 1 SD
Crenigacestat (LY3039478)	NCT02836600^84,105^	1	Active/not recruiting	Advanced solid tumors	≥20	25–50 mg PO TIW	1	1 SD

*BID* twice daily, *BIW* twice weekly, *DT* desmoid tumor, *GSI* γ-secretase inhibitor, *IV* intravenously, *PO* by mouth, *PR* partial response, *QD* once daily*, QW* once weekly, *Q2W* every two weeks, *SD* stable disease, *TIW* thrice weekly, *TBD* to be determined.

To date, nirogacestat (formerly PF-03084014) has been the most extensively studied GSI for the treatment of DT. Nirogacestat is a selective, noncompetitive, reversible GSI that has demonstrated antitumor activity in multiple, Notch-dependent preclinical models of disease^88,89^. In a phase 1 dose-escalation study (NCT00878189^90^), 64 patients with advanced solid tumors refractory to standard therapy received nirogacestat doses ranging from 20 to 330 mg BID. Among nine patients with DT (seven of whom were evaluable) included in the study, five patients demonstrated partial response by Response Evaluation Criteria in Solid Tumors (RECIST) v.1.0 while two patients experienced prolonged disease stabilization^86^. Drug-induced downregulation of Notch-related target protein HES4 also was established^86^. The most common treatment-emergent adverse events observed during the study were diarrhea, nausea, fatigue, hypophosphatemia, vomiting, rash, and decreased appetite, which were generally mild to moderate in severity. Long-term follow-up of the seven evaluable DT patients from this phase 1 trial showed that the five partial responders continued to maintain response lasting between 47.9 and 73.6 months, and only one patient experienced disease progression^91^.

In a phase 2 study (NCT01981551^92^), 17 patients with unresectable DT received nirogacestat 150 mg orally BID in 3-week cycles. Among 16 evaluable patients, five (29%) experienced partial response by RECIST v.1.1 criteria that was maintained for more than 2 years, while 11 experienced stable disease (with five patients experiencing prolonged stable disease for more than 2 years). There were no instances of disease progression^85^. All patients in this study experienced grade 1 and 2 adverse events, most commonly diarrhea (76%) and skin disorders (71%). The only grade 3 toxicity attributable to study drug was reversible hypophosphatemia, reported in eight patients (47%).

A report of four cases of pediatric and young adult patients with DT (three with FAP syndrome) who received nirogacestat on a compassionate use basis showed that after a median of 13.5 months of treatment (range 6–18 months), one patient experienced complete response, one patient experienced partial response, and one patient had stable disease, while one patient experienced disease progression after an initial partial response^87^. No grade 3 or 4 adverse events were reported; only one adverse event of grade 2 diarrhea was reported by one of the four patients.

Following from that report of four cases, a phase 2 trial (NCT04195399^93^) sponsored by the Children’s Oncology Group was initiated in September 2020 to evaluate nirogacestat in patients 12 months to 18 years of age with progressive, refractory DTs not amenable to surgery. The primary outcome measures are to estimate the 2-year progression-free survival rate and to describe the toxicities of nirogacestat in children and adolescents. Estimated study completion date is end of 2024.

In addition, the randomized, double-blind, placebo-controlled phase 3 DeFi study of nirogacestat 150 mg BID in ~142 adult patients with progressing DT (NCT03785964^94^) was initiated in May 2019. The primary endpoint of the study is progression-free survival as determined radiographically by RECIST v.1.1 criteria (confirmed by blinded, independent, central review), or clinical progression as assessed by the investigator (also confirmed by a blinded, independent central review). Secondary endpoints include objective response rate, and patient-reported outcomes including the Brief Pain Index short form (BPI-SF), **GO**under/**D**esmoid Tumor Research Foundation **DE**smoid **S**ymptom/Impact **S**cale (GODDESS), and European Organisation for Research and Treatment of Cancer Quality of Life Questionnaire–Core 30^95^. Results for the primary endpoint are expected in 2022.

AL101 (formerly BMS-906024) and AL102 (formerly BMS-986115) are structurally similar GSIs that are parenterally and orally administered, respectively, that inhibit activation of all four human Notch receptors^82,96^. Nonclinical experiments showed that AL102 demonstrated anti tumor activity against several solid tumor xenografts^96^. In a phase 1 dose-escalation study of AL101 (NCT01292655^97^), 94 patients with advanced solid tumors received weekly or bi-weekly intravenous doses of AL101. Of three patients with DT who were enrolled in the study, two experienced confirmed partial responses and one had stable disease as assessed by RECIST v.1.1 criteria^83,98^. The maximum tolerated dose was 4 mg QW with no dose-limiting toxicities in seven evaluable patients, and 6 mg Q2W with 1 dose-limiting toxicity in six evaluable patients (grade 3 diarrhea)^98^. Long-term follow-up of one patient from the phase 1 trial and a second patient enrolled in a compassionate use program reported confirmed partial responses (41 and 60% maximal tumor reduction from baseline) observed after 1.0 and 1.6 years of treatment with AL101, with response durations of 8.6+ and 2.6+ years, respectively^83^.

Similarly, in a phase 1 dose-escalation study of AL102 (NCT01986218^99^), 36 patients with advanced solid tumors received once daily or twice weekly oral doses of AL102. One patient with DT was enrolled in this trial and experienced stable disease as assessed by RECIST v.1.1 criteria for >6 months with maximum disease reduction of 16.5% after ~9 months of treatment^82^. All patients in this trial experienced treatment-emergent adverse events, most commonly diarrhea (72%), nausea (69%), hypophosphatemia (67%), fatigue (64%), decreased appetite (58%), and vomiting (53%).

The phase 2/3 RINGSIDE trial of AL102 in ~192 patients with progressive DT was initiated in March 2021 (NCT04871282^100^). Part A of this study is an open-label, dose-finding design in which AL102 will be administered at oral doses of 1.2 mg QD or 2–4 mg BIW, while part B will be conducted as a double-blind, placebo-controlled study using the optimal dose regimen identified in part A. The primary endpoint for the trial is progression-free survival, as assessed by RECIST v.1.1, or death by any cause. Secondary endpoints include overall response rate, duration of response, and patient-reported outcomes including BPI-SF, GODDESS, Patient-Reported Outcomes Measurement Information System (PROMIS)—physical function, and EuroQol 5-Dimensional Questionnaire (EQ-5D). The estimated completion date is first quarter of 2025.

Crenigacestat (formerly LY3039478) is a potent, oral, small molecule GSI that has been shown to inhibit Notch signaling in cell lines representing several different solid tumors and leukemia^101,102^. In a phase 1, open-label dose-escalation study in 110 patients with advanced or metastatic cancer (NCT01695005^103^), crenigacestat showed evidence of clinical activity in patients with breast cancer, leiomyosarcoma, and adenoid cystic carcinoma with gastrointestinal adverse events (diarrhea and nausea) most frequently reported^104^.

In another phase 1 study of crenigacestat (NCT02836600^105^), 11 Japanese patients with advanced solid tumors received either 25 or 50 mg crenigacestat three times weekly. One patient with DT in the 50 mg arm showed tumor reduction of 22% during the first 8 cycles of treatment and exhibited stable disease for 22.5 months^84^. Among all patients in the trial, the most frequently reported treatment-emergent adverse events were gastrointestinal (diarrhea, nausea, vomiting) that were generally mild to moderate in severity. Grade 3 hypophosphatemia was reported in two patients treated with 50 mg crenigacestat. There are currently no ongoing trials of crenigacestat in DT.

The GSIs are a drug class currently being evaluated in patients with progressive DT requiring systemic treatment. Once their role is established in that setting, it is likely GSIs will be evaluated in other settings including neoadjuvant or adjuvant treatment with loco-ablative therapies and/or in combinations with other agents to improve efficacy, overcome resistance, or decrease toxicity. Increasing insight into the clinical activity of GSIs in DT will better inform treatment choice and sequencing. Moving forward with combination strategies will depend on further understanding the different mechanisms of action and resistance associated with these agents and the potential identification of biomarkers that can predict response.

## Summary

Dysregulation of the Wnt signaling pathway plays a key role in DT pathogenesis, however, as the result of crosstalk, Wnt dysregulation may lead to activation of the Notch pathway. The overexpression of Notch pathway components in DT provides a rationale for development of pharmacologic agents targeting Notch. Results from preclinical and early-phase clinical trials suggest that inhibition of γ-secretase, which prevents the release of the NICD and its translocation to the nucleus, may be a promising therapeutic target for the treatment of DT. In phase 1 and phase 2 clinical trials to date, GSIs have been shown to be associated with durable antitumor activity in DT. Gastrointestinal adverse events appear to be most frequently associated with γ-secretase inhibition, although most were reported to be of mild to moderate severity. Results from later stage clinical trials of GSIs for the treatment of DT are anxiously awaited.

### Reporting summary

Further information on research design is available in the Nature Research Reporting Summary linked to this article.

## Supplementary information

NR Reporting Summary

## Data Availability

Source material for this review was derived from a search of the PubMed database in December 2021 for peer-reviewed, English language publications using the following terms: desmoid tumor, aggressive fibromatosis, desmoid-type fibromatosis, Wnt, β-catenin, *CTNNB1* mutation, *APC* mutation, Notch, γ-secretase, and γ-secretase inhibitor. Abstracts were reviewed to identify relevant pre-clinical and clinical studies characterizing the molecular mechanisms of DT pathogenesis, and results from clinical trials of GSIs in DT. Secondary references from selected articles also were reviewed. Finally, the ClinicalTrials.gov website was searched for completed and ongoing trials of GSIs for the treatment of DT.
